# One‐Step Exfoliation Method for Plasmonic Activation of Large‐Area 2D Crystals

**DOI:** 10.1002/advs.202204247

**Published:** 2022-09-14

**Authors:** Qiang Fu, Jia‐Qi Dai, Xin‐Yu Huang, Yun‐Yun Dai, Yu‐Hao Pan, Long‐Long Yang, Zhen‐Yu Sun, Tai‐Min Miao, Meng‐Fan Zhou, Lin Zhao, Wei‐Jie Zhao, Xu Han, Jun‐Peng Lu, Hong‐Jun Gao, Xing‐Jiang Zhou, Ye‐Liang Wang, Zhen‐Hua Ni, Wei Ji, Yuan Huang

**Affiliations:** ^1^ Advanced Research Institute of Multidisciplinary Science Beijing Institute of Technology Beijing 100081 P. R. China; ^2^ School of Physics and Key Laboratory of MEMS of the Ministry of Education Southeast University Nanjing 211189 P. R. China; ^3^ Institute of Physics Chinese Academy of Science Beijing 100190 P. R. China; ^4^ Department of Physics and Beijing Key Laboratory of Optoelectronic Functional Materials & Micro‐Nano Devices Renmin University of China Beijing 100872 P. R. China; ^5^ China North Vehicle Research Institute Beijing 100072 P. R. China; ^6^ Songshan Lake Materials Laboratory Dongguan 523808 P. R. China; ^7^ University of Chinese Academy of Sciences Beijing 100049 P. R. China

**Keywords:** mechanical exfoliation, photoluminescence, Raman spectrum, surface plasmonic polariton, two‐dimensional materials

## Abstract

Advanced exfoliation techniques are crucial for exploring the intrinsic properties and applications of 2D materials. Though the recently discovered Au‐enhanced exfoliation technique provides an effective strategy for the preparation of large‐scale 2D crystals, the high cost of gold hinders this method from being widely adopted in industrial applications. In addition, direct Au contact could significantly quench photoluminescence (PL) emission in 2D semiconductors. It is therefore crucial to find alternative metals that can replace gold to achieve efficient exfoliation of 2D materials. Here, the authors present a one‐step Ag‐assisted method that can efficiently exfoliate many large‐area 2D monolayers, where the yield ratio is comparable to Au‐enhanced exfoliation method. Differing from Au film, however, the surface roughness of as‐prepared Ag films on SiO_2_/Si substrate is much higher, which facilitates the generation of surface plasmons resulting from the nanostructures formed on the rough Ag surface. More interestingly, the strong coupling between 2D semiconductor crystals (e.g., MoS_2_, MoSe_2_) and Ag film leads to a unique PL enhancement that has not been observed in other mechanical exfoliation techniques, which can be mainly attributed to enhanced light‐matter interaction as a result of extended propagation of surface plasmonic polariton (SPP). This work provides a lower‐cost and universal Ag‐assisted exfoliation method, while at the same time offering enhanced SPP‐matter interactions.

## Graphical abstract

Here, the authors discover a universal Ag‐assisted exfoliation method, which can be used to directly integrate large‐scale 2D crystals with plasmonic nanostructures. The long propagation of surface plasmonic polariton in the hybrid structures results in extraordinarily photoluminescence enhancement for monolayer MoS_2_ and MoSe_2_. This technique allows to direct preparation and exploration of optical properties of emergent 2D crystals, which paves a new way for preparing large‐scale 2D materials and their future applications.

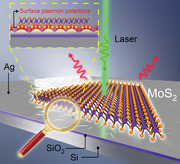

## 1 Introduction

2D materials and their heterostructures offer a unique platform for the exploration of different physical phenomena at the atomic‐scale limit, including quantum Hall effects,^[^ ^1^, ^2^ ^]^ moiré heterostructures related physics,^[^ ^3^, ^4^ ^]^ superconductivity,^[^ ^5^ ^]^ charge density waves (CDW)^[^ ^6^ ^]^ and magnetism.^[^ ^7^ ^]^ These intriguing properties are enabling diverse device applications such as field effect transistors,^[^ ^8^, ^9^ ^]^ quantum emitters,^[^ ^10^, ^11^, ^12^ ^]^ memory devices,^[^ ^13^ ^]^ optoelectronic devices,^[^ ^14^, ^15^, ^16^, ^17^ ^]^ and energy storage units.^[^ ^18^ ^]^ The continuous improvement of 2D material fabrication methods plays a crucial role in enabling the discovery of novel properties and device applications of 2D materials. Chemical vapor deposition (CVD) and mechanical exfoliation are the two most commonly used methods for obtaining mono‐ and few‐layers crystals. But despite CVD methods showing a clear advantage for preparing wafer‐scale monolayers,^[^ ^19^, ^20^ ^]^ or even twisted heterobilayers,^[^ ^21^ ^]^ the in‐plane strain, higher density of defects and impurities in CVD‐grown samples are still challenging to control.^[^ ^12^, ^22^, ^23^ ^]^

As one of the most widely used “top‐down” 2D materials fabrication strategy, mechanical exfoliation shows advantages in feasibility and cost‐effectiveness for exploring their novel properties. Traditional exfoliation methods via scotch tape or polydimethylsiloxane (PDMS) tape can be employed for getting high‐quality 2D monolayer crystals, but are limited in terms of sample size (≈10–100 µm) and exfoliation yield. An oxygen plasma cleaning method^[^ ^24^ ^]^ was proposed to enhance the yield and size of graphene and layered copper oxide high‐temperature superconductors. And more recently, Au‐enhanced exfoliation^[^ ^25^, ^26^, ^27^, ^28^ ^]^ offers a universal and one‐step approach to prepare large‐area 2D crystals. Although Au‐enhanced exfoliation shows obvious advantages for exploring many intrinsic physical properties of 2D crystals, for some optical measurements, especially PL spectroscopy, this method is severely hindered because of the quenching effect.^[^ ^25^, ^26^, ^28^, ^29^ ^]^ Au is also too costly for potential massive industrial production of 2D crystals. Another metal in IB group, silver (Ag), is significantly cheaper than gold, and this study explores in detail the feasibility of using Ag to replace Au to exfoliate 2D crystals, and other potential advantages beyond just cost saving.

In this work, to test the feasibility of Ag‐assisted exfoliation, we first perform theoretical calculations on the adhesive energy of different layered materials with silver. From there, we introduce a contamination‐free, one‐step, and universal Ag‐assisted mechanical exfoliation method, which has been successfully used to exfoliate 12 types of single‐crystalline monolayers with millimeter‐size, including metal‐dichalcogenides, black phosphorus (BP), 2D magnets and superconductors. The exfoliation efficiency of this Ag‐assisted method is almost identical to that of the Au‐enhanced exfoliation method. Interestingly, we also observed that the activated plasmon on the surface of Ag film is enhancing the PL emission of some exfoliated 2D semiconductors, like MoS_2_ and MoSe_2_, which is significantly higher when compared to the PL intensity generated from 2D semiconductors exfoliated on Au film and suspended ones. Our results demonstrate that the Ag‐assisted exfoliation method can provide a robust and lower‐cost approach to prepare emergent 2D materials, along with the capability to generate strong SPP field enhancement at the metal‐2D material interface.

## 2 Results and Discussion

### 2.1 Predication of Ag‐Assisted Exfoliation

**Figure 1:**
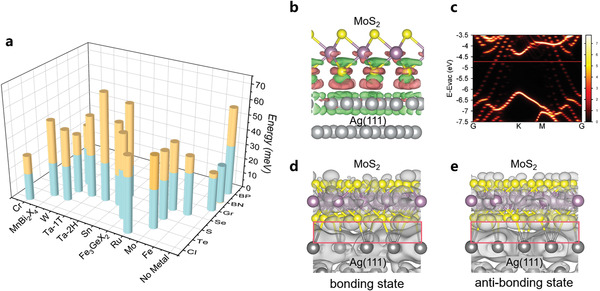
DFT calculations of 2D crystals on Ag substrate. a) Histogram of layered materials which shows the contrast of the interlayer binding energies (blue cylinders) and their adsorption energies on Ag (111) (yellow cylinders). The visible yellow cylinders represent the difference between the Ag/2D crystal and the interlayer interaction. b) DCD plots of Ag (111)/MoS_2_ interface. Isosurface value is 1 × 10^−3^ e Bohr^−3^. c) Unfolded band structure of Ag (111)/MoS_2_ interface. The red line represents the Fermi level. d–e) Isosurface contours of wavefunction norms for the bonding state and anti‐bonding state using an isosurface of 6 × 10^−5^ e Bohr^−3^.

Density functional theory (DFT) calculations were carried out to examine whether Ag thin film is a good candidate for assisted exfoliation, looking at specifically the relative adhesive energies and perturbation to the electronic structures of 2D layers. We used the ratio *R*_MA/IL_ to represent the layer‐Ag over the layer‐layer adhesive energies.^[^ ^28^ ^]^ **Figure** **1**a and Table S1 (Supporting Information) show the comparison of 18 representative 2D materials, which show an *R*_MA/IL_ range of 1.35–2.92 for 16 types of 2D crystals. Graphene and h‐BN are exceptions, with *R*_MA/IL_ values close to 1. Given the *R*_MA/IL_ values are significantly higher than 1 for most 2D crystals, with similar values to that of Au films, we expect substantial adhesion interactions between those 2D layers and the Ag surface.

The MoS_2_/Ag(111) interface is predicted to have an R_MA/IL_ value of 1.70, which suggests that the MoS_2_ crystal could be exfoliated using the Ag‐assisted method. However, to further understand the binding characteristics and to ensure the exfoliation material does not impact the intrinsic properties of the 2D material, one also needs to consider the strength of the perturbation when Ag is interacting with the electronic structures of the 2D layers. To this extent, we further examine the related characteristics of this representative interface. Figure 1b shows its differential charge density (DCD), from which significant covalent characteristics can be observed at the S/Ag interface, i.e., charge reduction near the interfacial atoms and charge accumulation between them. The binding energy of S/Ag (48 meV Å^−2^; 0.39 eV per unit cell) is slightly stronger than that of S/Au (40 meV Å^−2^; 0.35 eV per unit cell).^[^ ^28^ ^]^ Figure 1c shows the unfolded band structures of the MoS_2_/Ag interface, in which the shape and position of the valence and conduction bands of MoS_2_ are almost unchanged in comparison to those of the freestanding MoS_2_ monolayer. Therefore, the Ag thin film is even more conducive than the previously used Au thin film to maintain intrinsic electronic properties of those 2D materials laid on it.^[^ ^28^ ^]^ Figure 1d,e show the square of wave function norms of the fully occupied interfacial S‐Ag bonding and anti‐bonding states. Their energy levels are split by a few meV and depicts substantial interlayer attraction‐induced wave function overlap, known as covalent‐like quasi‐bonding (CLQB), at the MoS_2_/Ag interface. Given the sufficient 2D layer‐Ag adhesive energy and the nearly unaffected electronic structures, our theory predicts that the Ag surface is, in principle, capable of offering an effective exfoliation strategy.

### 2.2 Surface Roughness and Plasmon Activation of Ag Surfaces

To test whether the theoretical prediction is correct, we prepared Ag films with different thicknesses by thermal evaporation (details can be seen in Method part). Unlike evaporated Au that tends to form flat surfaces,^[^ ^28^ ^]^ evaporated Ag on a Si(111),^[^ ^30^ ^]^ sapphire^[^ ^31^ ^]^ or SiO_2_^[^ ^31^, ^32^ ^]^ substrate was demonstrated to have a rough surface, exhibiting nanoparticle‐like structures (Figure S1, Supporting Information). These connected particle‐like Ag can form nano cavities, which tend to promote the formation of localized‐surface‐plasmon‐resonance (LSPR),^[^ ^30^, ^31^, ^33^ ^]^ or propagating SPP.^[^ ^30^ ^]^ These two effects might substantially enhance PL intensities of several 2D semiconductor materials. A series of characterizations and simulations were thus conducted on the evaporated Ag thin films to unveil their plasmonic properties.

**Figure 2:**
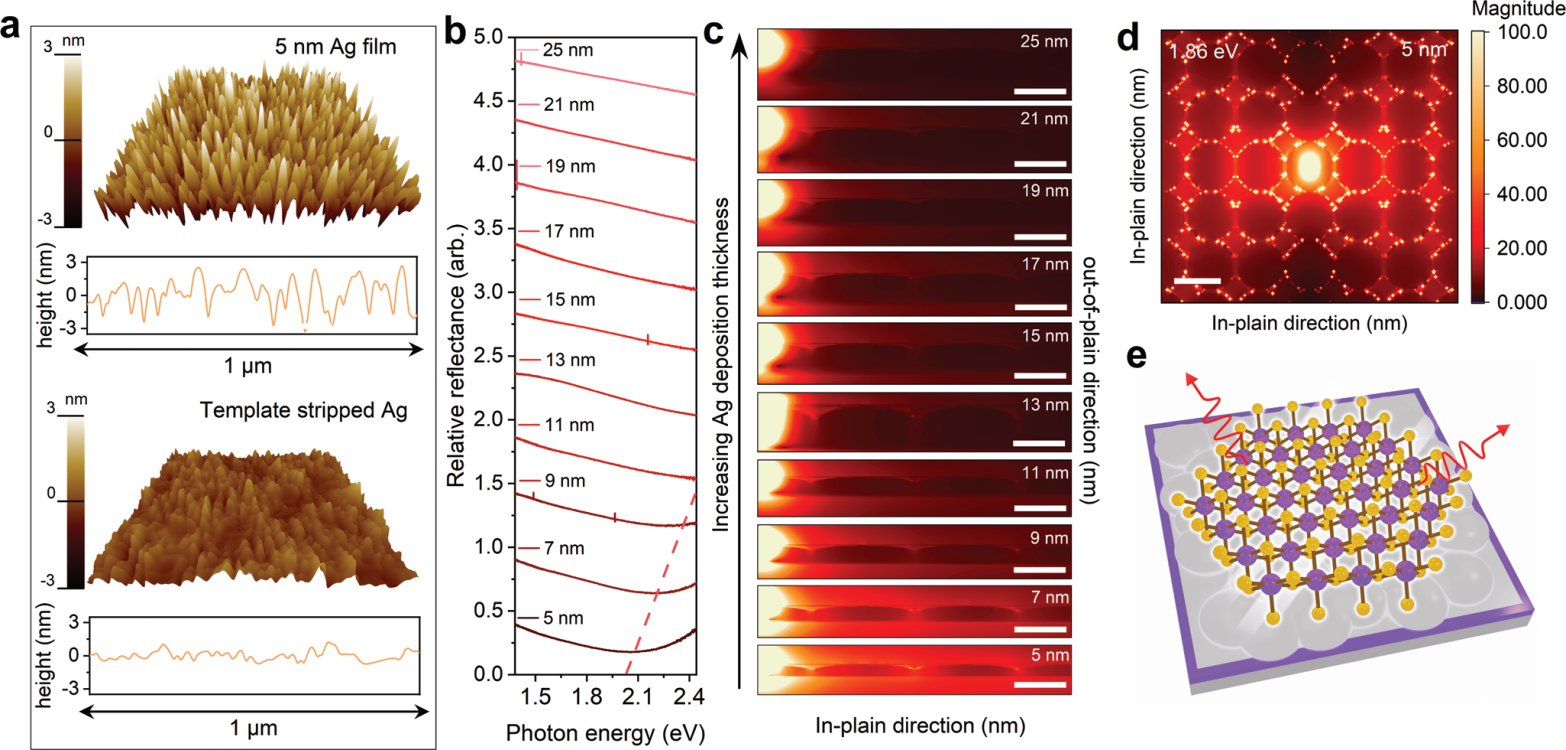
Surface and optical characterizations of Ag films. a) AFM of 5 nm Ag film surface (top) and TS Ag/epoxy/glass slide (bottom). b) Relative reflectance spectra ΔR/R of Ag films with varying thicknesses acquired in contrast to a self‐made silver mirror. Red dashed line denoting the position for plasmon resonance is employed for clarity. c) FDTD simulated cross‐sectional electric field intensity distribution of monolayer MoS_2_ supported by rough Ag film centered at 1.86 eV with thickness from 5 to 25 nm, the distance of SPP propagation along the *x*‐axis exhibit a conspicuous decline as thickness of Ag films increase. Scale bar is 40 nm. d) FDTD simulated top view electric field intensity distribution of monolayer MoS_2_ supported by 5 nm rough Ag film centered at 1.86 eV, indicating that the Ag films facilitate strong field confinement as well as long propagation of SPP. Scale bar is 80 nm. e) Schematic illustration of enhanced PL emission in a monolayer MoS_2_ exfoliated onto the surface of a 5 nm Ag film.

**Figure** **2**a depicts atomic force microscope (AFM) images of the surface morphology of an evaporated 5 nm Ag film (top) and another Ag film (bottom) prepared using the template‐stripping (TS) method (see Methods for details), respectively. The evaporated 5 nm Ag film shows a root‐mean‐square (RMS) roughness of 1.2 nm and the largest height variation is over 6 nm, while the surface prepared using the TS method yields a roughness of 0.4 nm, close to that of epitaxial‐grown Ag films on Si(111) (0.3 nm).^[^ ^30^ ^]^ Statistical analysis revealed that such a rough surface can be divided into surface particles with lateral sizes ranging from 40 to 100 nm (mean ≈80 nm), as a result of amorphous particle formation.^[^ ^28^ ^]^ We can attribute this “particle agglomeration” phenomenon to that Ag does not wet with Ti, which leads to Vollmer–Weber‐type growth and grain‐boundary pinning during Ag evaporation,^[^ ^32^, ^34^, ^35^ ^]^ which we directly illustrate in Figure S2, Supporting Information. As the deposition thickness increases from 5 to 25 nm, the surface morphology of those substrates shows similar characteristics in terms of particle shape, height, and lateral size (Figure S3, Supporting Information).

The surface of rough Ag films has been reported to show broad plasmon resonances.^[^ ^31^ ^]^ And when the exciton resonance matches with their plasmon resonances, PL intensity of supported samples will be enhanced. Relative reflectance spectra (ΔR/R) of the surfaces of those thin films were acquired to probe the plasmon resonance, where the reflectance is normalized to a reflective silver mirror. As shown in Figure 2b, the 5 nm Ag films showed a broad plasmon resonance at ≈2.1 eV, which can potentially be excited by a 532 nm laser source, and excitons generated in 2D crystals like monolayer MoS_2_ and WS_2_ (A‐exciton resonances at ≈1.86 and ≈1.99 eV, respectively). For thicker substrates, their plasmon resonances generally experience a blue shift, which suggests that SPP excited in thicker Ag films can hardly couple with their exciton resonances. Furthermore, as calculated by finite‐difference time‐domain (FDTD) method (detailed modeling see Methods), Figure 2c,d shows the cross‐sectional and top view of field intensity distribution of Ag films centered at A‐exciton resonance of monolayer MoS_2_, respectively. For Ag films thinner than 9 nm, strong field confinement as well as long propagation of SPP (200 nm for 5 nm Ag films) can be achieved, indicating possible PL enhancement via Purcell factor^[^ ^31^ ^]^ and exciton‐reexcitation,^[^ ^30^ ^]^ respectively. As Ag thickness increases, the propagation of SPP in in‐plane directions decreases significantly (Figure 2d and Figure S4, Supporting Information) and become negligible for films thicker than 9 nm. Our simulation shows that PL intensity of some 2D semiconductors, such as monolayer MoS_2_ and WS_2_, on thinner Ag films could be strongly enhanced (schematic shown in Figure 2e). The enhancement, however, does start to decline as Ag thickness increases. The enhanced PL intensity suggested by simulation is confirmed with experimental results, and will be discussed in detail in the following sections.

### 2.3 Exfoliation of Plasmonic Activated 2D Monolayer Crystals

**Figure 3:**
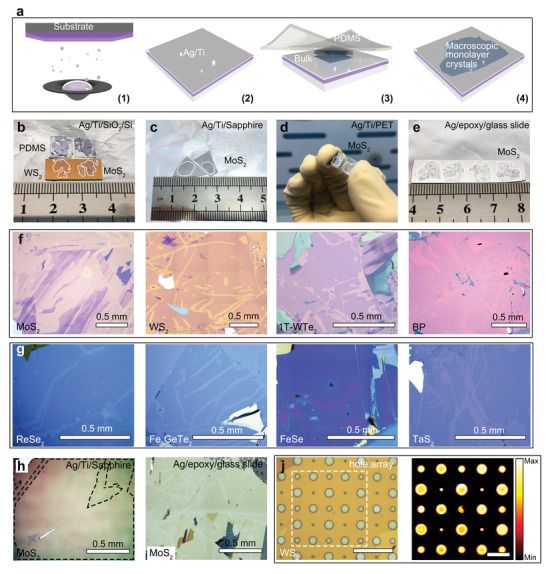
Ag‐assisted exfoliation procedures and optical characterization of exfoliated samples. a) Schematic illustration of the exfoliation procedures. b) Exfoliated macroscopic MoS_2_ and WS_2_ on 15 nm Ag film supported by SiO_2_/Si substrates, and bulk crystals on PDMS tapes. c) Exfoliated macroscopic MoS_2_ supported by sapphire substrates. d) Exfoliated MoS_2_ supported by elastic PET substrates. e) Exfoliated MoS_2_ on Ag/epoxy/glass slide substrate. (f) Optical microscope images of some 2D crystals exfoliated on 15 nm Ag film, including MoS_2_, WS_2_, 1T‐WTe_2_, and BP. g) Optical microscope images of exfoliated millimeter size 2D crystals on 5 nm Ag film, including ReSe_2_, Fe_3_GeTe_2_, FeSe, and TaS_2_. h–i) Optical microscope images of exfoliated millimeter size MoS_2_ on sapphire substrate and TS Ag, respectively. j) Optical microscope and PL mapping images of exfoliated monolayer WS_2_ on 15 nm Ag film with hole array, the scale bars in the two images are 40 and 20 µm, respectively.

Based on theoretical and simulation results, we experimentally explored using Ag films as an exfoliation medium for layered materials. Here, 12 representative 2D monolayers were successfully prepared using this Ag‐assisted exfoliation method. **Figure** **3**a shows the schematic illustrations of Ag‐assisted exfoliation, which was also tested and confirmed to be compatible on various substrates, such as SiO_2_/Si (Figure 3b), sapphire (Figure 3c), PET (Figure 3d), and quartz surfaces. Those monolayers can also be exfoliated onto Ag surfaces using the TS method (Figure 3e, detailed preparation is discussed in Methods), which indicates that surface roughness is not a critical issue for successful exfoliation (height profiles of exfoliated samples shown in Figure S5, Supporting Information). Notably, the yield of monolayer drastically drops after exposure of the as‐prepared Ag surface to air in 10 s (Figure S6, Supporting Information), indicating the ambient atmosphere condition is detrimental to Ag‐assisted exfoliation (most likely oxidation).

Figure 3f,g, and Figures S7 and S8 (Supporting Information) illustrate those 12 exfoliated monolayers, including MoS_2_, MoSe_2_, MoTe_2_, WS_2_, WSe_2_, ReS_2,_ and BP (semiconductors), 1T'‐WTe_2_ and FeSe (superconductors), Fe_3_GeTe_2_ and MnBi_2_Te_4_ (magnets) and TaS_2_ (CDW), which show the macroscopic size and high uniformity. Furthermore, the presented method behaves similarly to Au‐enhanced exfoliation in that it does not require the substrate to be fully covered by Ag. This enables direct exfoliation^[^ ^36^ ^]^ of free‐standing 2D monolayers using patterned hole‐array substrates. Figure 3j shows an optical microscope image and a corresponding PL mapping of a large‐scale suspended WS_2_ monolayer. The suspended WS_2_ monolayer presents much stronger PL emission on hole areas than those supported areas. Additionally, low wavenumber Raman modes of 2D crystals suspended and supported were acquired, the suppression of breathing and shearing Raman modes for samples on Ag films implies the existence of CLQB as demonstrated in Figures S9 and S10, Supporting Information. This CLQB‐induced pinning effect is also found in Au/2D materials interfaces.^[^ ^36^, ^37^ ^]^

### 2.4 Observation and Mechanism of PL Enhancement

**Figure 4:**
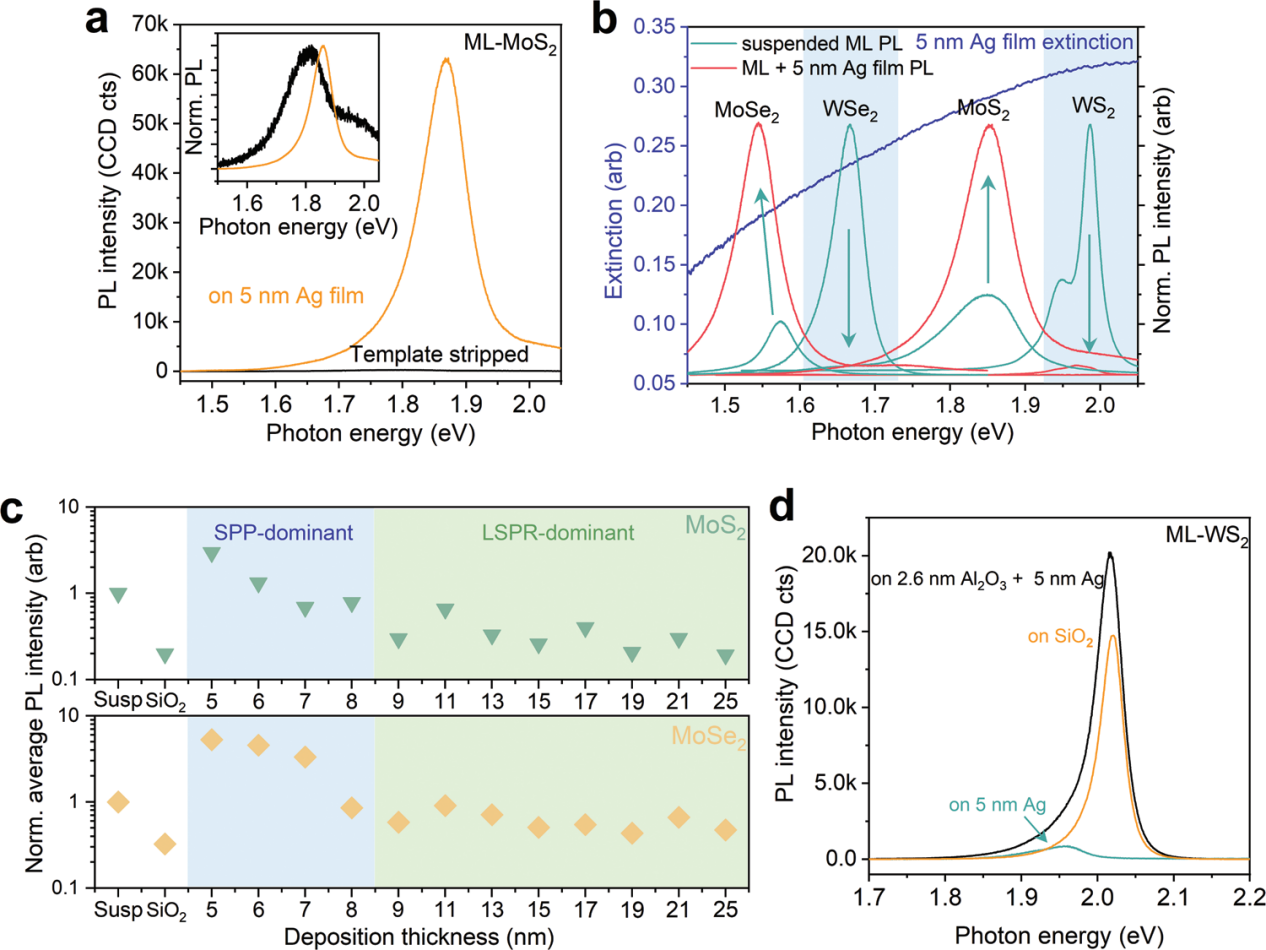
PL behavior of four exfoliated TMDC semiconductors on Ag film. a) PL spectra of monolayer MoS_2_ exfoliated onto 5 nm Ag films and TS Ag substrates. Inset: normalized PL spectra of monolayer MoS_2_ exfoliated onto 5 nm Ag films and TS Ag. b) Extinction spectrum of 5 nm Ag film (blue) and normalized PL intensity of monolayer MoS_2_, MoSe_2_, WS_2_, and WSe_2_ on 5 nm Ag films (orange) and on hole arrays (green). c) The normalized average PL intensity of as‐exfoliated monolayer MoS_2_ and MoSe_2_ on Ag films with different deposition thickness and SiO_2_/Si substrates, normalized to PL intensity of their respective suspended monolayers, each average intensity data was acquired at five different spots. Generally, PL emission of Ag‐supported samples experience a decrease with increasing Ag thickness. d) PL spectra of monolayer WS_2_ on 5 nm Ag film (green), on SiO_2_/Si substrate (orange), and 2.6 nm Al_2_O_3_ + 5 nm Ag film (black), which indicates PL quenching is mainly caused by charge transfer.

For most 2D semiconductors, PL intensities of SiO_2_‐supported monolayers are typically weaker than those of suspended monolayers,^[^ ^38^ ^]^ which are further suppressed in metal‐supported monolayers due to additional charge transfer^[^ ^39^ ^]^ from the metal surfaces. As discussed above, the yield of exfoliated 2D flakes is not sensitive to the roughness of Ag. However, the roughness strongly affects the plasmonic properties of exfoliated 2D monolayers. As expected, no appreciable PL intensity was observed on the TS Ag film‐supported MoS_2_ monolayer because of the flat metallic surface (black, **Figure** **4**a). To our surprise, an extraordinarily prominent A‐exciton emission located at ∼1.86 eV (PL intensity and peak position mapping shown in Figure S11, Supporting Information) was observed on the rough surface Ag supported monolayer (orange, Figure 4a). The PL intensity of monolayer MoS_2_ on the rough Ag film is 3.1, 11, and over 200 folds stronger than those of suspended, SiO_2,_ and TS Ag supported monolayers, respectively. Figure 4b summarizes the comparison of PL spectra for suspended and rough Ag surface‐supported MoS_2_, MoSe_2_, WS_2,_ and WSe_2_ monolayers. Similarly, the PL emission of plasmonic coupled MoSe_2_ is also 4.8 times than that of the suspended MoSe_2_. The enhanced PL of MoS_2_ and MoSe_2_ are fairly air‐stable with only 24.2% (MoS_2_) and 17.7% (MoSe_2_) decline after exposing to ambient conditions for 10 days (Figure S12, Supporting Information). However, unlike those molybdenum‐based samples, the supported WS_2_ and WSe_2_ monolayers on rough Ag film do suffer from significant PL quenching. Their PL intensities are 1/24 and 1/620 times those of WS_2_ and WSe_2_ monolayers exfoliated onto the SiO_2_/Si substrate, respectively. All PL measurements were conducted under the same circumstances, details see Methods.

The three orders of magnitude variation of PL intensity is, most likely, a result of the competition between exciton‐plasmon coupling activated PL enhancement^[^ ^31^, ^40^ ^]^ and Ag induced quenching effect.^[^ ^31^, ^41^, ^42^ ^]^ The exciton‐plasmon coupling usually leads to the propagation of SPP^[^ ^30^ ^]^ so that the exciton‐SPP‐photon conversion and exciton re‐excitation could enhance PL intensity for 2D semiconductors. An additional effect lies in the enhancements of incident excitation (near‐field emission) and excitonic emission (spontaneous emission), which are positively‐related to the local photonic densities^[^ ^31^, ^33^, ^43^ ^]^ and highly dependent on the surface morphology of Ag films (e.g., roughness).^[^ ^31^, ^33^, ^44^ ^]^

We examined the thickness‐dependent competition of these two competing effects by exfoliating MoS_2_ and MoSe_2_ samples on a series of Ag films in a range of thickness from 5 to 25 nm. Figure 4c presents thickness‐dependent normalized PL intensities of monolayer MoS_2_ (MoSe_2_) on the series of Ag films (those for WS_2_ and WSe_2_ were shown in Figure S13, Supporting Information), in which the strongest PL was found on the 5 nm sample. The intensity clearly decays from 5 to 8 nm and remains weak for thicker ones. The critical thickness of 8 nm is comparable with the simulated critical thickness of 9 nm for propagating SPP,^[^ ^30^ ^]^ supporting its dominant role in strengthening the PL intensity of MoS_2_ on Ag films thinner than 8 nm. For Ag layers thicker than 9 nm, although the propagation of SPP is suppressed, the thickness‐independent LSPR still contributes toward the PL intensity and provides a small intensity enhancement.^[^ ^31^ ^]^

For WS_2_ monolayer, its Fermi level sits at −4.87 eV,^[^ ^45^ ^]^ over 0.6 eV lower than that of the Ag(111) surface (−4.3 eV), giving rise to a n‐type doping to WS_2_ at the WS_2_/Ag interface. The *n*‐doping promotes the formation of negatively‐charged trions and thus lowers its PL quantum yield,^[^ ^29^, ^30^, ^32^, ^46^, ^47^ ^]^ which is even more efficient in high quantum yield materials (e.g., monolayer WS_2_ and WSe_2_).^[^ ^31^, ^42^ ^]^ To verify this effect, a 2.6 nm Al_2_O_3_ dielectric layer was deposited by atomic layer deposition onto the 5 nm Ag film, where a flake of monolayer WS_2_ was then transferred. As expected, the PL emission of monolayer WS_2_ on 5 nm Ag with the Al_2_O_3_ dielectric layer is 1.3 times stronger than the sample on the SiO_2_/Si substrate (Figure 4d), which confirms that PL quenching primarily originates from electron transfer of Ag to WS_2_. Such quenching effect is also expected for other high work function monolayers, e.g., WSe_2_.

## 3 Conclusion

We have presented a universal Ag‐assisted exfoliation technique that creates a unique integration between large‐scale 2D crystals and plasmonic nanostructures, while offering multiple advantages over Au‐enhanced exfoliation. The effectiveness and universality of this technique are justified by theoretical calculations as well as spectroscopy results, which confirmed the strong interactions caused by CLQB between Ag and 2D materials, exceeding the interlayer van der Waals interactions. Optical characterizations were conducted immediately after exfoliation, showing enhanced PL emissions of monolayer MoS_2_ and MoSe_2_, as a result of SPP propagation enhanced light‐matter interaction. The high quality and yield of as‐exfoliated samples prove that our technique is not only important for basic research of emergent 2D materials, but also shows great potential in industrial production of ultrathin 2D crystals. This study provides an alternative option for direct exfoliation and optical characterization of emergent 2D crystals. We expect this SPP‐induced PL enhancement could extend to other low quantum yield 2D materials with luminescent exciton resonances in the range of plasmon resonances of respective Ag films. Whether large‐scale 2D crystals can be directly exfoliated onto other metal substrates for functionalities such as surface‐enhanced Raman scattering^[^ ^48^ ^]^ or fano resonances,^[^ ^49^ ^]^ or even insulating substrates where their intrinsic physical properties are well kept and readily to be explored worth further investigations.

## 4 Experimental Section

### DFT Calculations

DFT calculations were performed using the generalized gradient approximation for the exchange‐correlation potential, the projector augmented wave method,^[^ ^50^, ^51^ ^]^ and a plane‐wave basis set as implemented in the Vienna ab initio simulation package (VASP).^[^ ^52^ ^]^ Van der Waals interactions was performed at vdW‐DF level for all calculations, with the optB86b functional for the exchange potential.^[^ ^53^ ^]^ The energy cutoff for the plane‐wave basis‐sets was set to 700 eV for variable volume structural relaxation of pure materials and 500 eV for invariant volume structural relaxation of 2D materials on Ag (111) surface. A *k*‐mesh of 9 × 9 × 1 was adopted to sample the first Brillouin zone of the conventional unit cell of the Ag (111) slab. The mesh density of *k*‐points was kept fixed when calculating other structures. Seven layers of Ag atoms, separated by an 18 Å vacuum region, were employed to model the Ag (111) surface. In geometry optimization, all atoms in the supercell except for the last four layers of Ag atoms were allowed to relax until the residual force per atom was less than 0.01 eV Å^−1^. To make the electronic properties more accurate, lattice parameters of 2D materials were fixed and those of the Ag (111) surface slab were changed, with the lattice mismatch between 2D layer and the Ag (111) surface being kept lower than 4.5%. DCD was calculated using Δ*ρ*_DCD_ = *ρ*_All_ − *ρ*_Ag_ − *ρ*_2D_. Here *ρ*_All_ is the total charge density of the 2D layer/Ag (111) interface, while *ρ*_Ag_ and *ρ*_2D_ are the total charge densities of the individual Ag surface and the 2D layer, respectively. Unfolded band structure was calculated using the KPROJ program based on the k‐projection method.^[^ ^54^, ^55^ ^]^

### FDTD Simulations

The electric field distribution was calculated by finite difference time domain method. The defined dielectric constant of Ag was from Johnson and Christy (n+ik where *n* = 0.051585 and *k* = 3.9046).^[^ ^56^ ^]^ Semi‐spheroid Ag nanoparticles (5 nm in height and 80 nm in lateral size) placed on Ag films with different thicknesses were used to mimic the surface morphologies of as‐deposited Ag films and to qualitatively analyze the propagation of SPP. A 0.6 nm thick monolayer MoS_2_ asymmetric thin film (in‐plane and out of plane dielectric constants are 2.22 and 1.70) was placed on top of the Ag nanoparticles, and an in‐plane dipole was placed at the center of the film to mimic the effect of an exciton.

### Ag‐Assisted Mechanical Exfoliation

The SiO_2_/Si substrate was first treated with pure oxygen plasma or air plasma for approximately 5 min, and at a power of 40 W and 50 sccm flow for cleansing the substrate surface of contaminations and ensuring better adhesion between substrate and metal layer. Plasma irradiation can also increase nucleation centers to enhance the roughness of Ag films. The metal layer deposition was carried out in an in‐glovebox thermal evaporation system (VNano). An ultrathin adhesive layer (3 nm Ti or Cr) was first evaporated (0.3 Å s^–1^) on SiO_2_/Si substrate (100 nm oxide), followed by deposition of Ag film (1 Å s^–1^). After metal deposition, an as‐cleaved surface of bulk crystals on PDMS (Gel‐Pak) tape was brought into contact with the substrate. After gently pressing the PDMS tape vertically for ≈1 min, uniform contact can be ensured and the tape can then be instantly removed from the substrate. Millimeter size or even bare eyes observable monolayers can thus be obtained with ultraclean surfaces, whose sizes are merely restrained by the size of their bulk crystals. Nonetheless, exfoliation yield still can be severely hindered if the adhesive is too thin. Once the Ti layer is less than 1 nm and Ag layer is less than 5 nm, the yield of monolayer drops steeply. This is probably because 1 nm Ti is not a continuous film that can no longer offer sufficient adhesion with the support below. In this case, evaporated Ag/Ti can be easily peeled off from the substrates. With optimized 15 nm Ag/3 nm Ti films, the success rate of exfoliation is nearly unity.

The bulk crystals are mainly bought from commercial companies (2D Semiconductors and HQ Graphene), and some of them are supplied by other groups. All of these crystals can be exfoliated into large‐scale monolayers with size dependent on the size of our bulk crystals. The thickness of bulk crystals is one critical factor to our exfoliation. Once the bulk crystal is too thick (thicker than 0.5 mm), its rigidness will influence a fine contact between itself and the substrate, which decreases or even eliminates the size of yielded monolayers. There is no need to cleave the bulk crystals too thin because they might be reduced into small fragments. The optimized bulk crystals should be continuous and thin, yet not transparent if observed against light illumination. This exfoliation is not fitted for ambient conditions that the exposure to humidity and oxygen can cause fast oxidation of surficial Ag, and consequently loses all adhesion ability. However, it could be well performed in a glovebox with humidity and oxygen concentration <0.01 ppm, where the as‐prepared adhesive layer can last for several days and still keep its effectiveness. It has been also tested that under 100 ppm oxygen concentration, as‐deposited Ag films lose its functionality in roughly 6 hours.

For preparation of suspended samples, SiO_2_/Si wafer was first patterned by UV lithography (Karl Süss MA6), and then hole arrays were opened up by reactive ion etching (CF_4_/O_2_) with 10 µm diameter and 10 µm depth. After that, ultrathin Ag and Ti were deposited in an in‐glovebox thermal evaporation system, where the same Ag‐assisted exfoliation technique illustrated above was conducted.

In order to find other exfoliation media layer, we also tested copper (Cu), chromium (Cr), and titanium (Ti) for exfoliation of several 2D crystals. The tested layered crystals include MoS_2_, WS_2,_ and BP. However, the flake sizes are very small and the yield is quite low, similar as the 2D flakes exfoliated on bare SiO_2_ substrates.

### Template Stripping of Ag Films

A 150 nm Ag was first evaporated in an in‐glovebox thermal evaporation system onto a sacrificial silicon substrate at 1 Å s^–1^ rate to reduce the possibility of grain boundary formation. The wafer was then cut into specified sized pieces and coated with epoxy adhesive. Glass slides were then pressed onto the epoxy so that the epoxy could cover their whole substrate surfaces. Afterward, glass slide/epoxy/Ag/SiO_2_/Si stacks were heated up on a hotplate at 60 °C for 2 h. The glass slide/epoxy/Ag could be easily peeled off from the SiO_2_/Si sacrificial layer, which yielded a considerably smooth Ag surface.

### Optical and Surface Characterizations

The Raman and PL measurements were operated on a WITec alpha300R system equipped with a wavelength 532 nm diode‐pumped solid‐state laser and power at 0.8 mW (if not otherwise noted). The low wavenumber Raman detection capability of this device was >7 cm^–1^. The silicon Raman mode at 520.7 cm^–1^ was used for calibration before measurements.

Surface characterizations were carried out using AFM (Oxford, Asylum Research Cypher S, and Park XE15) in a tapping mode.

## Conflict of Interest

The authors declare no conflict of interest.

## Author Contributions

Q.F., J.Q.D., and X.Y.H. contributed equally to this work. Y.L.W., Z.H.N., W.J., and Y.H. are equally responsible for supervising this discovery. Y.H., W.J., and Q.F. conceived the project. J.Q.D., Y.H.P., and W.J. performed DFT calculations. Q.F. prepared exfoliated 2D crystals, and performed optical measurements. Q.F., J.P.L and, L.L.Y. acquired extinction spectra of as‐deposited Ag films, and performed FDTD simulations. Q.F., X.Y.H, X.H., and Z.Y.S. measured the surface morphology of as‐deposited Ag films and TS Ag films by AFM. Q.F., Y.Y.D., Y.H., W.J., J.Q.D., L.L.Y., X.J.Z., and Y.H.P. analyzed the data, wrote the manuscript, and all authors discussed and commented on it.

## Supporting information

Supporting Information

## Data Availability

The data that support the findings of this study are available from the corresponding author upon reasonable request.
