# Supplementary material for: One‐Step Exfoliation Method for Plasmonic Activation of Large‐Area 2D Crystals
Source: Adv Sci (Weinh). 2022 Sep 14;9(32):2204247. doi: 10.1002/advs.202204247 (PMC9661865; doi:10.1002/advs.202204247)
Supplement: Supplementary file 1 — Supporting Information [file ADVS-9-2204247-s001.pdf]

ADVANCED SCIENCE
Open Access

Supporting Information

for *Adv. Sci.*, DOI 10.1002/advs.202204247

One-Step Exfoliation Method for Plasmonic Activation of Large-Area 2D Crystals

Qiang Fu, Jia-Qi Dai, Xin-Yu Huang, Yun-Yun Dai, Yu-Hao Pan, Long-Long Yang, Zhen-Yu Sun, Tai-Min Miao, Meng-Fan Zhou, Lin Zhao, Wei-Jie Zhao, Xu Han, Jun-Peng Lu, Hong-Jun Gao, Xing-Jiang Zhou, Ye-Liang Wang, Zhen-Hua Ni*, Wei Ji* and Yuan Huang**

Supporting Information for

One-step exfoliation method for plasmonic activation of large-area 2D crystals

Qiang Fu#, Jia-Qi Dai#, Xin-Yu Huang#, Yun-Yun Dai, Yu-Hao Pan, Long-Long Yang, Zhen-Yu Sun, Tai-Min Miao, Meng-Fan Zhou, Lin Zhao, Wei-Jie Zhao, Xu Han, Jun-Peng Lu, Hong-Jun Gao, Xing-Jiang Zhou, Ye-Liang Wang, Zhen-Hua Ni*, Wei Ji* and Yuan Huang**

#These authors contributed equally to this work.

*Correspondence to: yeliang.wang@bit.edu.cn (Y.L.W.), zhni@seu.edu.cn (Z.H.N.), wji@ruc.edu.cn (W.J.), and yhuang@bit.edu.cn (Y.H.)

Table of Contents

1. Surface morphology characterizations for Ag and Au films. (Figure S1-S3)

2. FDTD simulation of electromagnetic field distributions of monolayer MoS_2/Ag film hybrid structures. (Figure S4)

3. AFM and optical images, Raman, PL and electrical characterizations for the exfoliated 2D materials, heterostructures and suspended 2D materials on Ag film. (Figure S5-S14)

4. Calculated energies of 2D materials supported by Ag(111). (Table S1)

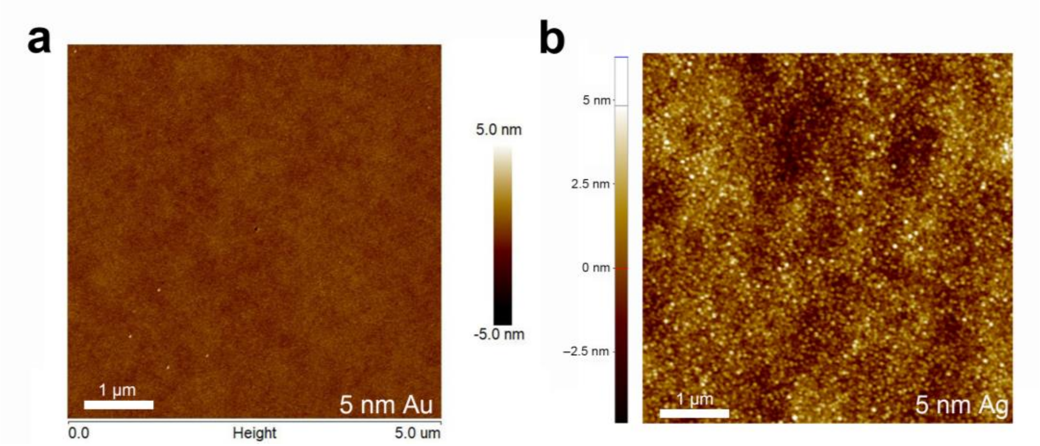

Figure S1 (a-b) AFM images of 5×5 µm area 5 nm Au/2 nm Ti film (a) and 5 nm Ag/2 nm Ti film on 300 nm SiO_2/Si substrate. Ag films evaporated onto Ti show rough nanoparticle-like surfaces.

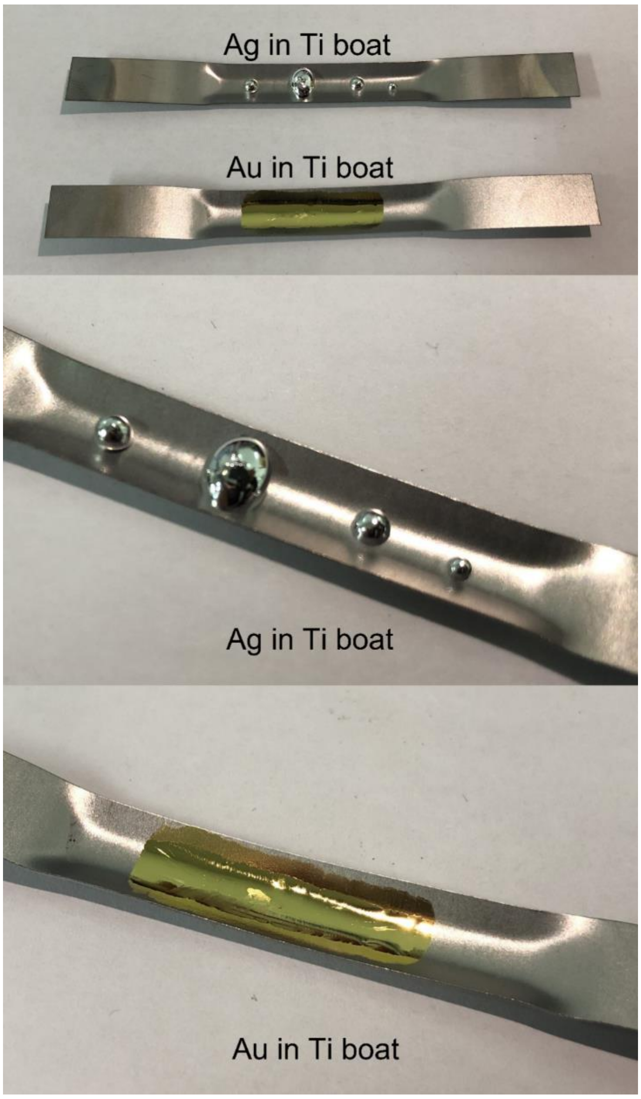

Figure S2 Au and Ag in tungsten boat covered with 5 nm thick evaporated Ti after solidification from liquid states. The balled-up Ag implies obvious dewetting between Ag and Ti, whereas Au wets Ti and tends to form uniform films. Their dewetting is responsible for highly-rough Ag surfaces.

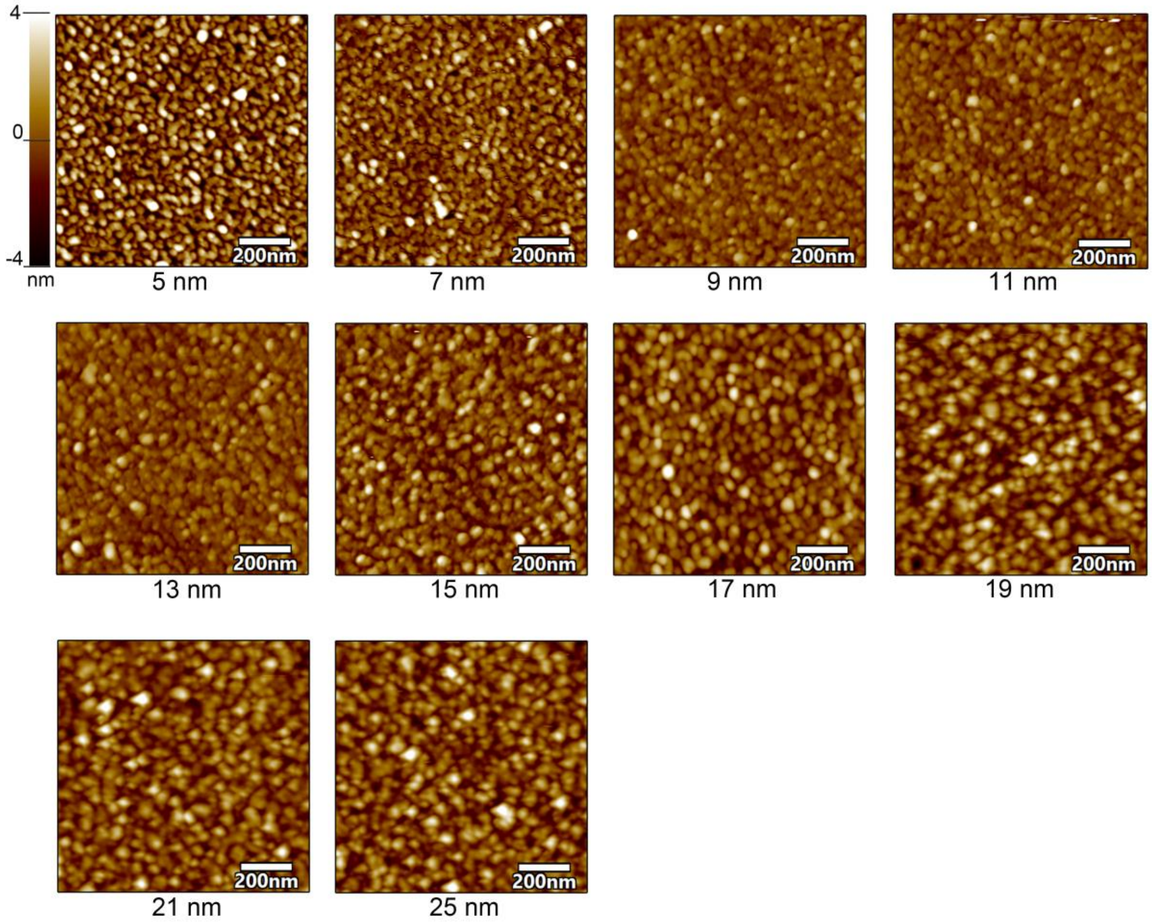

Figure S3 AFM images showing the surface morphology of Ag films with increasing thickness, as the deposition thickness increases, the surface morphology barely changes. Their RMS roughness (Ag film thickness) are 1.295 nm (5 nm), 0.948 nm (7 nm), 0.772 nm (9 nm), 0.765 nm (11 nm), 0.783 nm (13 nm), 0.862 nm (15 nm), 0.829 nm (17 nm), 0.953 nm (19 nm), 0.822 nm (21 nm), and 0.910 nm (25 nm), respectively. Despite their roughness fluctuate because of difference in particle densities, morphologies and sizes of nanoparticles are nearly identical in these Ag films.

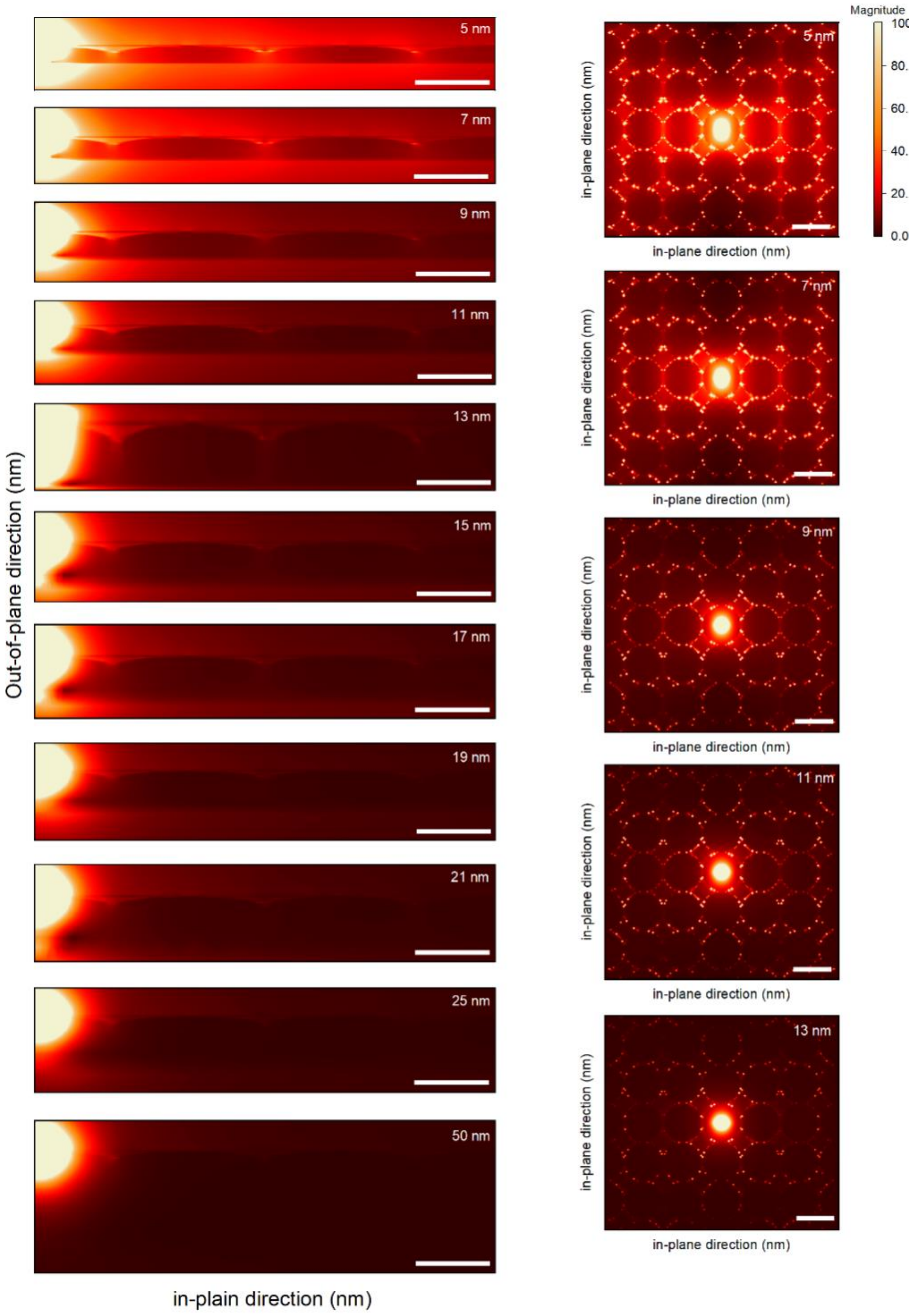

Figure S4 FDTD simulation of electromagnetic field distribution in the in-plane and out-of-plane direction of monolayer MoS_2/Ag film hybrid structures with different deposition thickness for photon energy at 1.86 eV (A exciton emission of monolayer MoS_2), scale bars are 40 nm and 80 nm, respectively. The propagation of SPP severely decreases with increasing Ag deposition thickness, indicating less photons are coupled into the SPP modes.

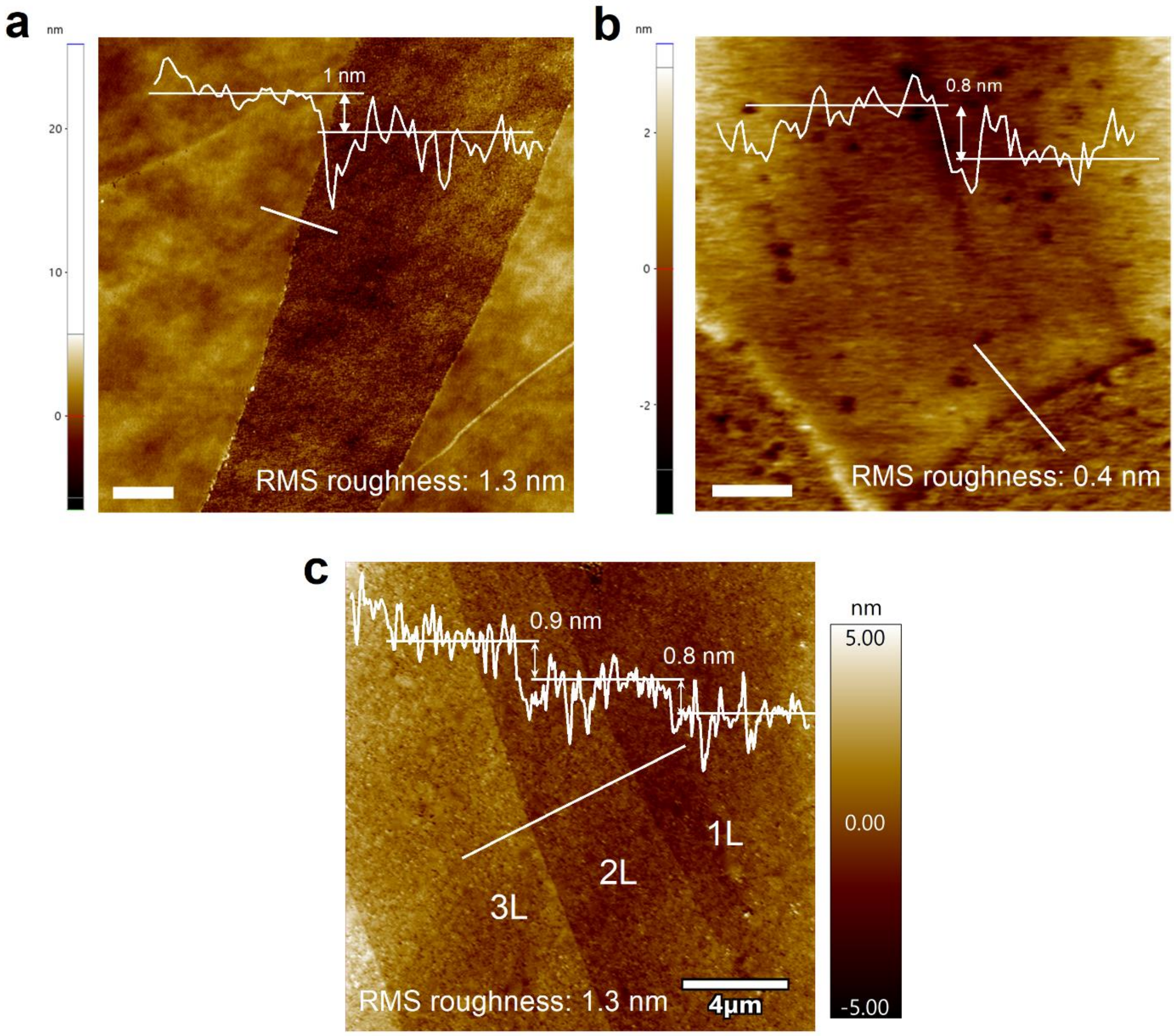

Figure S5 Morphologies and height profiles of Ag exfoliated MoS_2. (a-b) Monolayer MoS_2 exfoliated onto 5 nm Ag film (a) and TS Ag (b) with RMS roughness of 1.3 nm and 0.4 nm, respectively. Scale bar is 5 μm. (c) Monolayer to 3-layer MoS_2 exfoliated onto 5 nm Ag film. Despite highly rough surfaces of Ag films, layer number can still be distinguished by AFM.

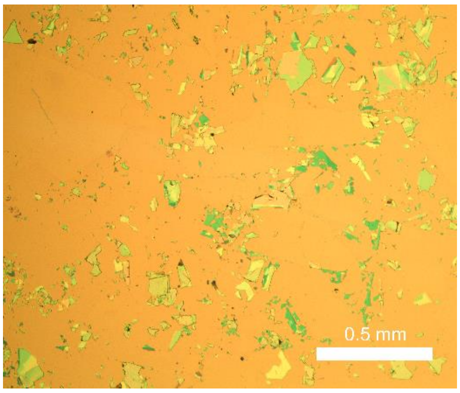

Figure S6 Optical microscope image of MoS_2 exfoliated onto 15 nm thick Ag after exposing to air for approximately 10 seconds, no macroscopic monolayers can be found.

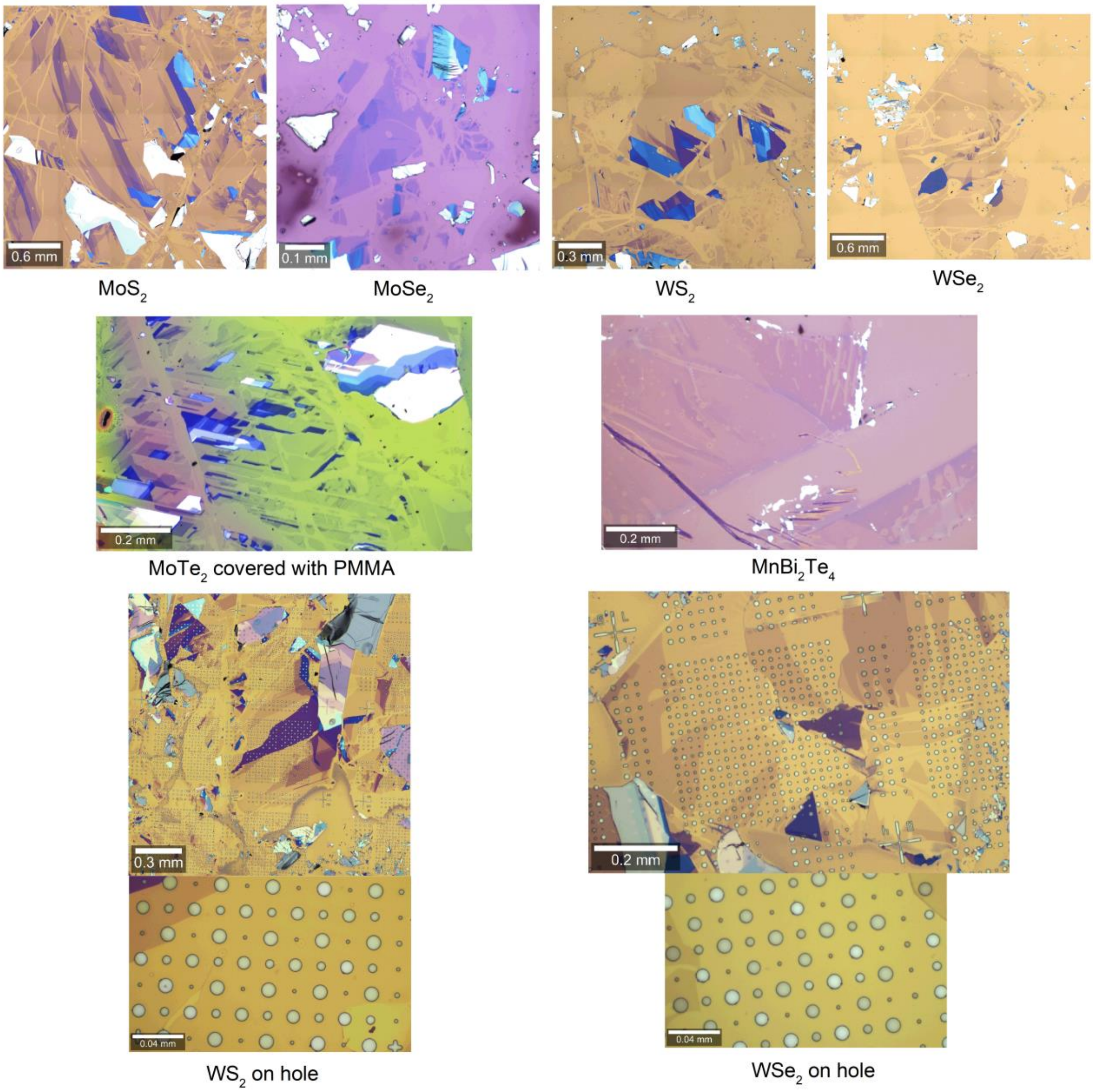

Figure S7 Optical microscope images of large-scale monolayer or few-layer MoS_2, $MoSe_2$, WS_2, WSe_2, $MoTe_2$ and $MnBi_2Te_4$ exfoliated onto Ag and patterned Ag films.

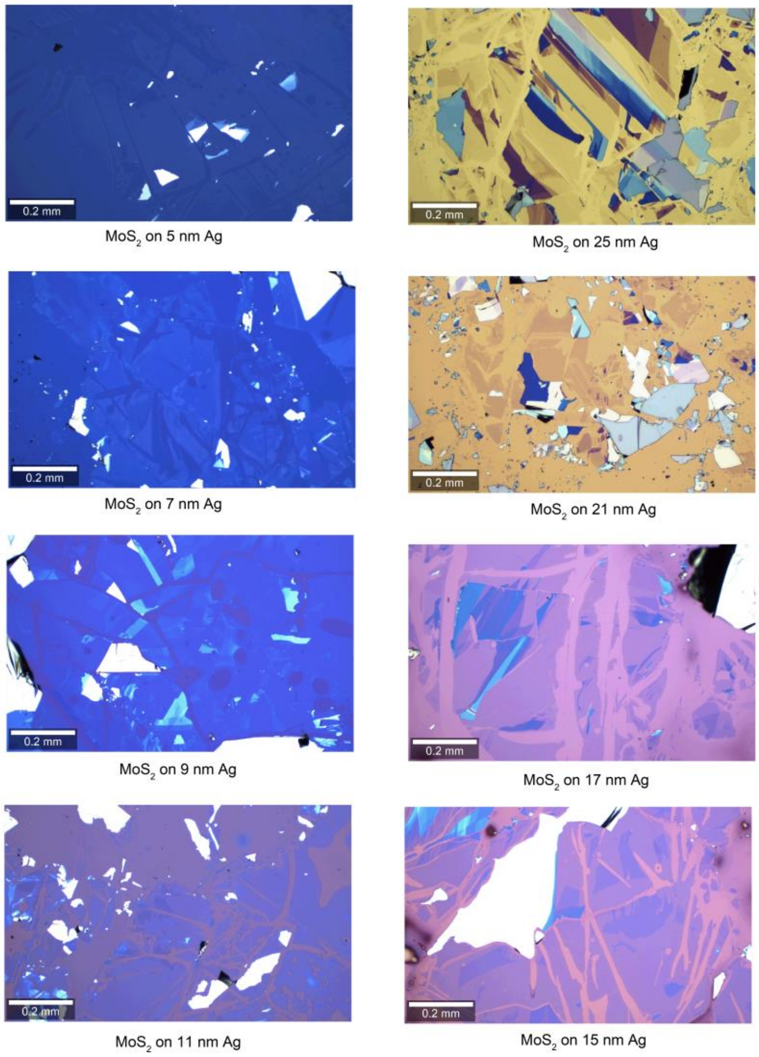

Figure S8 Optical microscope images of large-scale monolayer or few-layer MoS_2 exfoliated onto Ag film with varying thickness.

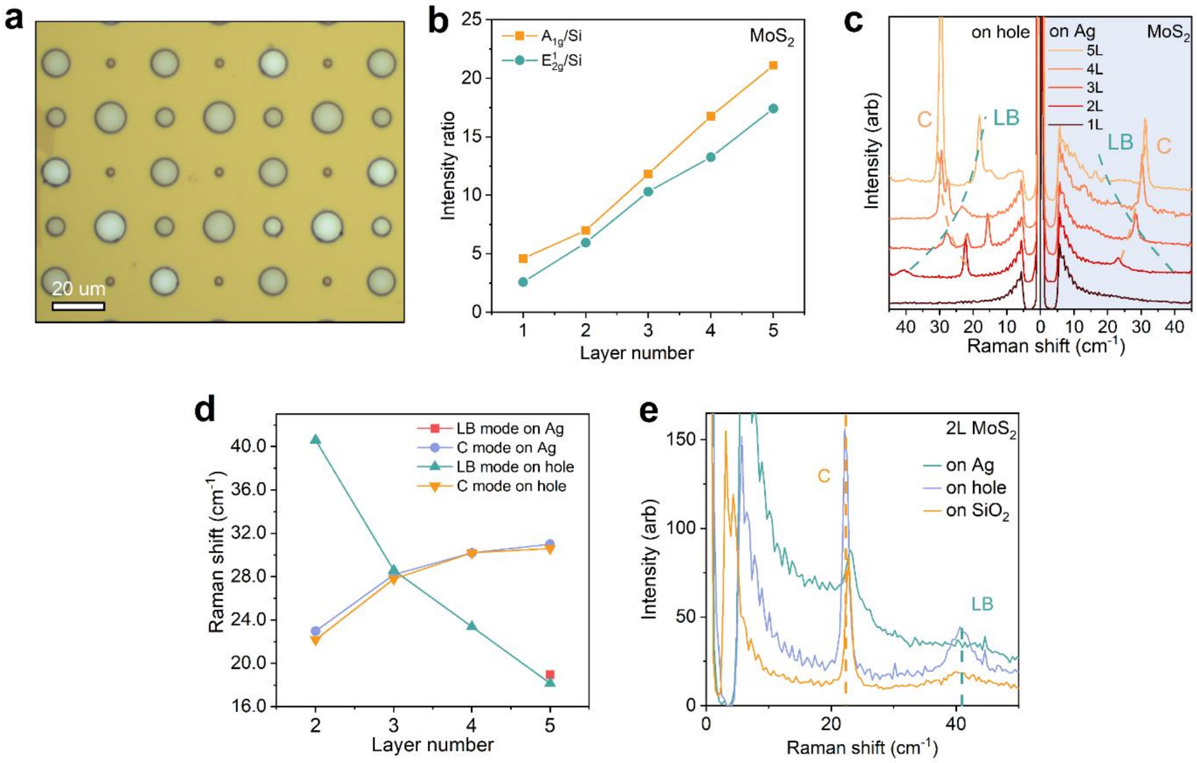

Figure S9 Raman spectra of 2D crystals on Ag film. (a) Optical microscope image of as-exfoliated free-standing monolayer MoS_2 on 15 nm Ag film. (b) Evolution of intensity ratio between characteristic Raman modes of 1L to 5L MoS_2 on 15 nm Ag films and Si around 520.1 cm^{-1}. (c) Comparison between 1L to 5L MoS_2 exfoliated onto hole arrays (left) and onto 15 nm patterned Ag film (right), suppression of interlayer vibrational modes implies strong interactions between Ag layer and as-exfoliated samples. (d) Peak positions of LB and C modes derived from (c), LB modes of 2L to 4L MoS_2 on Ag are not presented because they cannot be distinguished. (e) Comparison of LW Raman modes of 2L MoS_2 on Ag, hole and SiO_2/Si, dashed lines are marked for shearing mode (C) and layer breathing mode (LB), respectively.

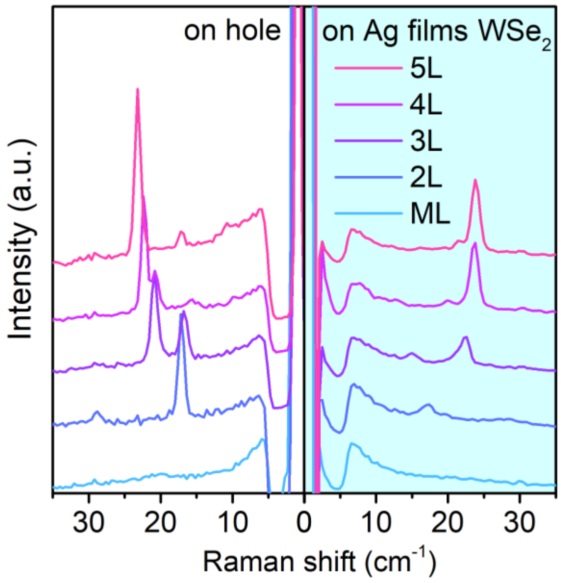

Figure S10 Comparison of LW Raman activated modes between 1L to 5L WSe_2 exfoliated onto hole arrays (left) and onto 15 nm Ag films (right), suppression of interlayer vibrational modes implies strong interactions between Ag films and as-exfoliated samples.

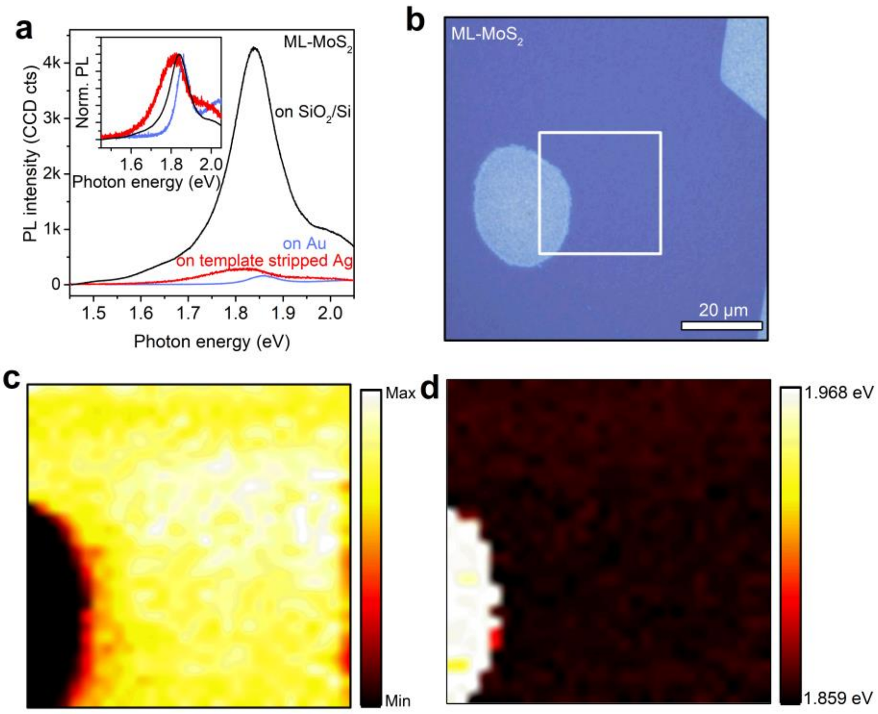

Figure S11 (a) Comparison of PL intensity of monolayer MoS_2 exfoliated onto 5 nm Au (red), template stripped Ag (blue), and SiO_2/Si (black) substrates. (b) Optical microscope image of as-exfoliated monolayer MoS_2 on 5 nm APSs, the surface roughness is observable to the bare eye, rectangle area denotes the 30 x 30 μm mapping area. (c) PL intensity mapping of the as-exfoliated monolayer MoS_2, extraordinarily strong PL is nearly uniform across the whole area. (d) PL peak position mapping of as-exfoliated monolayer MoS_2, which is almost invariable at ~1.86 eV.

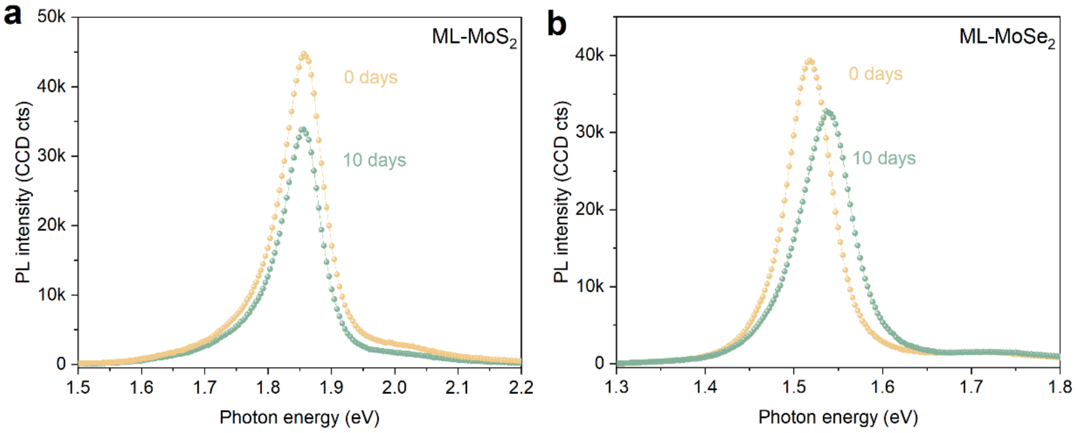

Figure S12 PL spectra and morphologies of exfoliated samples. (a-b) Strongest PL spectra of monolayer MoS_2 (a) and $MoSe_2$ (b) exfoliated onto 5 nm Ag films directly after exfoliation (yellow) and exposed to ambient conditions for 10 days (green). Their PL intensities decayed for 24.2% and 17.7%, respectively.

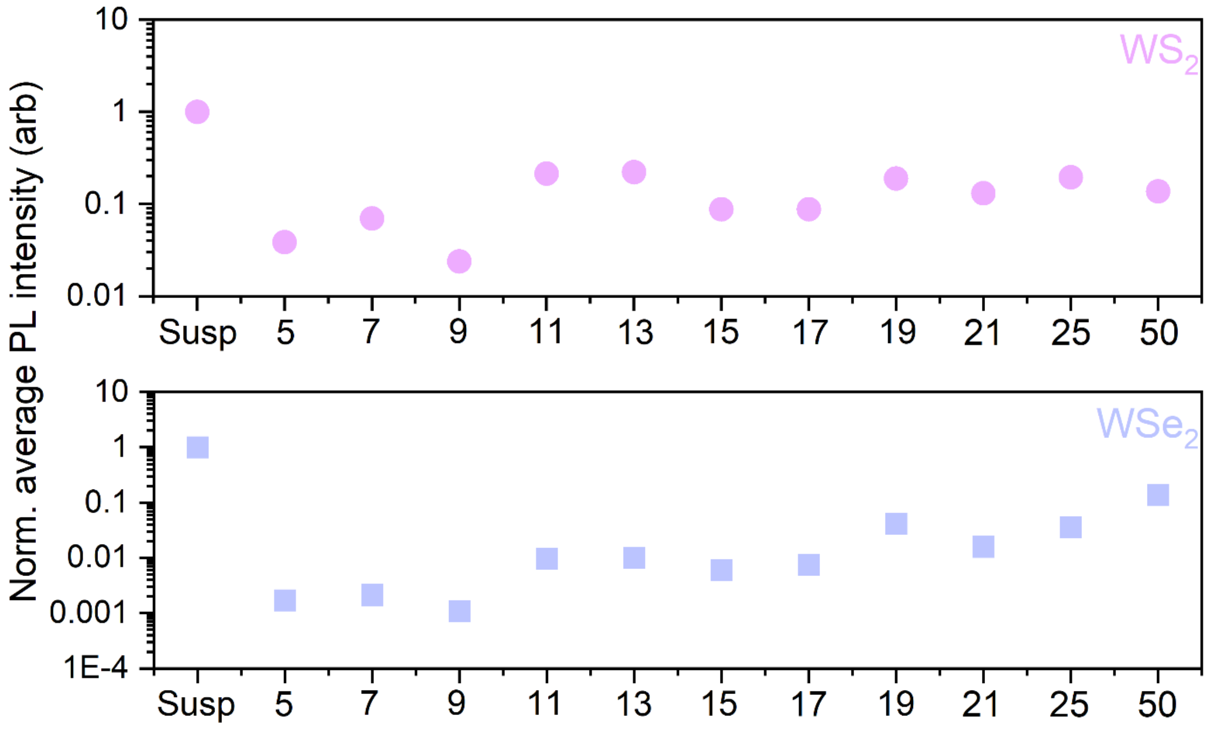

Figure S13 Normalized average PL intensity of as-exfoliated monolayer WS_2 and monolayer WSe_2 on Ag films with increasing deposition thickness, normalized to PL intensity of their respective suspended monolayers, each average intensity data point was the average intensity at 5 different spots.

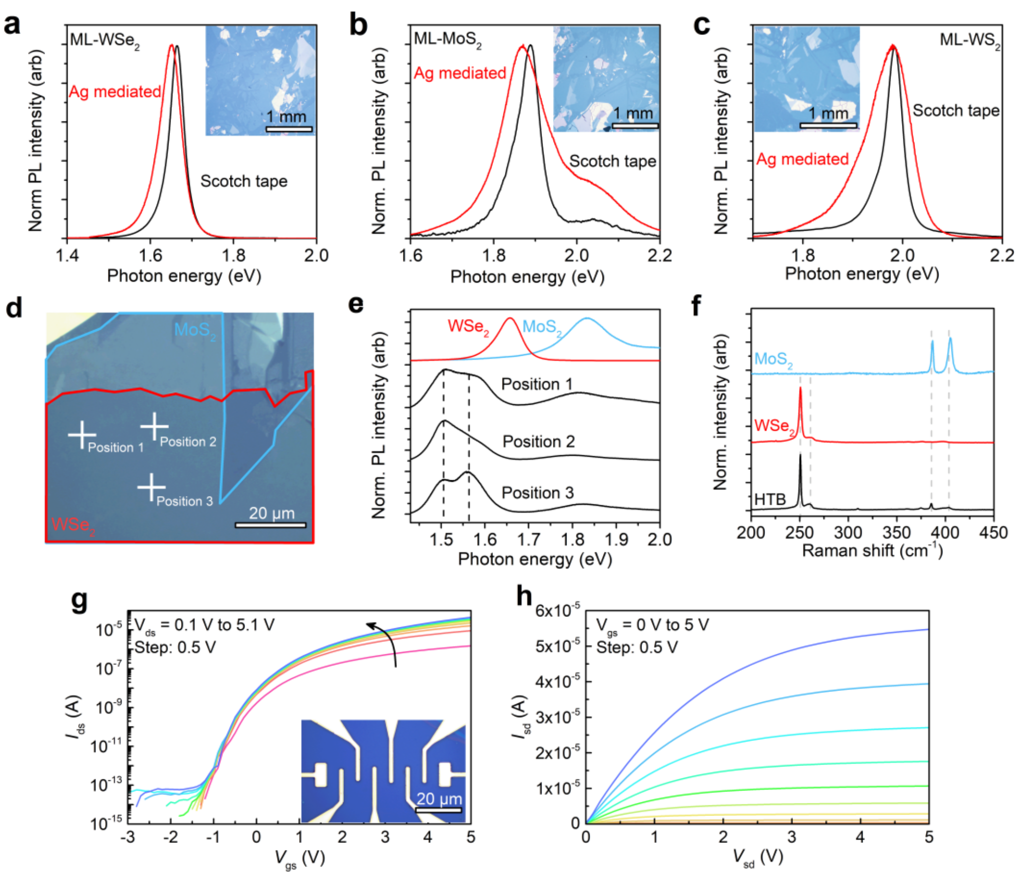

Figure S14 Optical and electrical characterization of monolayer and heterobilayer samples transferred onto SiO_2/Si substrates. (a-c) PL spectra comparison for monolayer WSe_2, monolayer MoS_2, and monolayer WS_2 respectively, prepared via Ag-mediated exfoliation (red) and scotch tape method (black). (d) Optical microscope image of a MoS_2/WSe_2 heterobilayer. (e) Room temperature PL spectra of MoS_2/WSe_2 heterobilayer at 3 different sites of the overlap region and intralayer exciton emission at single layer regions. Two lower energy peaks emerged in the heterobilayer PL spectra that is unobservable in the single layer regions are considered IX emission. (f) Raman spectra of a MoS_2/WSe_2 heterobilayer acquired at the single layer and overlap regions in (d), dashed gray lines are for visual clarity. (g) Transfer characteristic of a back-gated monolayer MoS_2 FET for V_{ds} from 0.1 V to 5.1 V, step: 0.5 V, from which mobility ~15 $cm^2\ V^{-1}\ s^{-1}$ and on-off ratio ~10^8 are

derived. h Output characteristic for the same device for V_{gs} from 0 V to 5 V, step 0.5 V.

Supplementary note 1: Raman evidence of CLQB between 2D crystals and Ag films

Theoretical results indicate that the interaction between many layered crystals and Ag atoms is stronger than the intrinsic crystal interlayer van der Waals interactions. This interaction between Ag and layered crystal is similar to the CLQB interaction between Au interface and layered crystals[26, 28]. For exfoliated 2D crystals on Ag film, in particular the bottom layers that have direct contact with Ag, the CLQB at the interface is expected to suppress or even extinct some of the low wavenumber (LW) interlayer Raman modes, resembling a pinning-like effect as presented in our previous work[37]. For layered materials such as MoS_2 and WSe_2, their Raman activated interlayer modes are in-plane E_{2g}^2 shearing mode (C mode) and out-of-plane B_{2g}^2 intralayer breathing mode (LB mode).

Figure S9a presents an optical microscope image of as-exfoliated MoS_2 2D crystals on Ag film patterned with hole arrays, the layer number can be easily distinguished from the optical contrast, and also confirmed by intensity ratio between characteristic Raman peak (A_{1g} and E_{2g}^1) and Si peak (~520.7 cm^{-1}) as shown in Figure S9b. Figure S9c shows LW Raman activated modes of suspended (left) and supported MoS_2 on Ag film (right), and the layer number of MoS_2 range from monolayer to 5L. The evolution of peak positions for C and LB modes are summarized in Figure S9d. In comparison to previous reports[1-3], Raman shift of C mode and LB mode in suspended and supported MoS_2 show no major difference. However, there exhibits conspicuous suppression or extinction of multiple LW modes for MoS_2 on Ag film, especially for LB mode that cannot be distinguished even up to 4L, which supports the existence of CLQB at the MoS_2/Ag interface. Since the back-scattering setup also extinct or suppress LW Raman modes because substrates can hinder vibrations, samples directly exfoliated onto SiO_2/Si substrates were taken as reference, which can only weakly couple with 2D crystals via van der Waals interactions. As demonstrated in Figure S9e, despite clear quenching in LW Raman modes, LB mode of 2L MoS_2 on SiO_2/Si substrate is still weakly distinguishable at ~40.03 cm^{-1}, which supports our speculation about the CLQBs induced pinning-effect at the interface.

Supplementary note 2: Transfer and characterization of exfoliated large-scale samples

Resembling the traditional wet transfer of 2D crystals, polymethyl methacrylate (PMMA) can be used as transfer media, and NaI/I_2 solution as a mild etchant to remove underlying Ag films without posing damage to the samples. However, organic residues will contaminate the sample surface, which is detrimental to establishing a good electrical contact or carrying out different types of surface characterizations. Here, we replace the Ti adhesive with a water-soluble polyvinylpyrrolidone (PVP) layer. Ag films carrying monolayer samples can detach from the sacrificial substrate in less than 10 mins if immersed into DI water. Detached Ag films are subsequently fished out by a glass slide and then cleaned for three times in DI water. The target substrates then pick up the Ag films in DI water, which is then heated up, cleaned, and etched by NaI/I_2 for approximately 6 hours to eliminate the Ag film. In order to be certain the optical properties of Ag-mediated samples are well preserved during the exfoliation and transfer procedures, monolayer MoS_2, WS_2 and WSe_2 are prepared via Ag-mediated exfoliation and traditional scotch tape method. Samples on Ag films are subsequently transferred onto another SiO_2/Si substrate for PL measurement. Figure S14a-c shows the PL spectra of these monolayer samples on SiO_2/Si substrate prepared by Ag-mediated exfoliation (red) and scotch tape method (black), which demonstrate high quality of our Ag-mediated samples as the spectral shapes and peak positions are almost identical. The notable difference in emission peak width is likely resulted from the variable intensity ratio between neutral exciton, B exciton, and trion emission, which is reasonable given the fluctuations in the quality and defect densities of raw bulk crystals. Insets of Figure S14a-c are optical microscope images of transferred samples, which remains intact and their sizes well preserved, making them available for multiple purposes such as stacking of type II heterobilayers (HTBs) for investigation of interlayer exciton (IX) emission or the fabrication of nano devices such as FETs.

For manual stacking of HTBs, if the interlayer twisted angle is not specifically demanded, alignment procedures under a transfer station is unnecessary due to extraordinarily large area of our transferred samples. Following the same process, another detached and cleaned Ag film containing 2D crystals just needed to be successively fished out by the previous substrate. By this method, monolayer MoS_2/monolayer WSe_2 type II HTBs are prepared, and subsequently PL and Raman spectroscopies are conducted. As shown in Figure S14e, the room temperature PL spectra of MoS_2/WSe_2 HTB contains three major peaks, the two lower energy peaks

around 1.50 and 1.56 eV can be considered as IX emission peaks. These peaks corresponds to the transition between electrons in K valleys of MoS_2 and holes resulted from the hybridized states at Γ points, which is not detectable in either monolayer MoS_2 or monolayer WSe_2. The Raman spectrum of HTB is also observable for characteristic intralayer vibrational modes of both monolayer films. These results demonstrate that fine interface quality and interlayer coupling has been perfectly established in our manually stacked HTBs, enabling potential investigations of optical and vibrational properties in manually stacked twisted or well aligned heterostructures and homostructures.

For the purpose of demonstrating device application potentials of our exfoliated and transferred samples, we fabricated a back-gated FET device based on the transferred monolayer MoS_2. Standard photolithography process, followed by thermal evaporation and liftoff were employed to define the 50 nm Au/5 nm Ti electrodes (inset of Figure S14g). Transfer and output characteristic curves are shown in Figure S14g and S14h, respectively, from which large on-off ratio ~10^8 and carrier mobility ~20 $cm^2\ V^{-1}\ s^{-1}$ can be derived, which is comparable to most monolayer MoS_2 back-gated devices. The electrical transport measurements indicate that the electrical properties are well preserved during the transfer procedures.

Supplementary note 3: PVP functionalized transfer of as-exfoliated samples and heterobilayer stacking

A wafer-scale SiO_2/Si substrate was first spin-coated with PVP solution (PVP powder Mol. wt. 38000 10% wt. in 1:1 ethanol/acetonitrile solution, 3000 rpm 30s, acceleration 2000 rpm/s twice and baked at 150 °C for 5 min). 150 nm Ag film was then deposited in an in-glove-box thermal evaporation system at 1 Å s^{-1} rate. After the sample exfoliation, the substrates carrying Ag films and samples are placed upside down and then immersed into DI water to dissolve the PVP layer. After roughly 10 min, the Ag films with samples will be detached from the substrate and then fished out by glass slides, which is subsequently cleaned for three times with DI water. After that the target substrates were used to fish out the Ag films and is then heated on a hotplate at 60 °C to evaporate the liquid underneath. Afterwards, substrates with Ag film were cleaned by oxygen plasma for 2 min to remove possible PVP residues, and immersed into I_2/NaI solution (2.5 g I_2 and 10 g NaI in 100ml DI water) for approximately 6 hours to remove the Ag films. Substrates carrying large-scale monolayers are then cleaned successively by acetone, isopropanol, and DI water to eliminate potential ion residues. Stacking of heterobilayers started with the transfer of as-exfoliated samples by the same PVP functionalized transfer method illustrated above. Monolayer samples were successively transferred onto a new SiO_2/Si substrate, each time followed by a lift-off and annealing process to remove interfacial organic residues and to ensure that good interlayer adhesion and coupling were established. Since our samples were macroscopic in size, no specific alignment procedure was needed, as long as interlayer twisted angle does not need to be specified.

Supplementary Table 1. Calculated energies of 18 considered 2D materials

2D materials	Ads. Energy on Ag(111) (eV per unit cell)	Interlayer Coupling Energy (eV per unit cell)	Ads. Energy on Ag(111) (eV Å-2)	Interlayer Coupling Energy (eV Å-2)	$R_{MA/IL}$	Magnetic Structure*
Graphene	0.134	0.118	0.0255	0.0225	1.13	NM
h-BN	0.134	0.136	0.0246	0.0250	0.99	NM
P(Black)	0.840	0.484	0.0583	0.0334	1.74	NM
$CrCl_3$	0.854	0.550	0.0279	0.0180	1.55	FM
$RuCl_3$	0.776	0.574	0.0461	0.0368	1.35	NM
MoS_2	0.389	0.229	0.0448	0.0263	1.70	NM
$MoSe_2$	0.426	0.243	0.0453	0.0258	1.76	NM
$MoTe_2$	0.509	0.281	0.0474	0.0261	1.82	NM
FeSe	0.515	0.381	0.0391	0.0285	1.37	NM
$SnSe_2$	0.795	0.275	0.0625	0.0214	2.92	NM
SnS_2	0.641	0.243	0.0550	0.0207	2.66	NM
WS_2	0.350	0.227	0.0395	0.0261	1.51	NM
WTe_2	1.033	0.516	0.0474	0.0235	2.02	NM
$MnBi_2Te_4$	0.812	0.368	0.0510	0.0229	2.23	FM
WSe_2	0.385	0.241	0.0409	0.0256	1.60	NM
2H-TaS_2	0.683	0.249	0.0716	0.0262	2.73	NM
1T-TaS_2	0.528	0.278	0.0543	0.0286	1.90	NM
Fe_3GeTe_2	0.739	0.434	0.0556	0.0327	1.70	FM

*NM: Non-magnetic; FM: Ferromagnetic

Reference

1. Huang, X., L. Zhang, L. Liu, Y. Qin, Q. Fu, Q. Wu, R. Yang, J.-P. Lv, Z. Ni, L. Liu, W. Ji, Y. Wang, X. Zhou, and Y. Huang, Raman spectra evidence for the covalent-like quasi-bonding between exfoliated MoS2 and Au films. *Sci. Chin. Info. Sci.* **64**, 140406 (2021).

2. Zeng, H., B. Zhu, K. Liu, J. Fan, X. Cui, and Q. M. Zhang, Low-frequency Raman modes and electronic excitations in atomically thin MoS2 films. *Phys. Rev. B* **86**, 241301 (2012).

3. Zhang, X., W. P. Han, J. B. Wu, S. Milana, Y. Lu, Q. Q. Li, A. C. Ferrari, and P. H. Tan, Raman spectroscopy of shear and layer breathing modes in multilayer MoS2. *Phys. Rev. B* **87**, 115413 (2013).
